# Spatial pattern of the ecological environment in Yunnan Province

**DOI:** 10.1371/journal.pone.0248090

**Published:** 2021-06-22

**Authors:** Dali Wang, Wenli Ding

**Affiliations:** 1 Faculty of Geography, Yunnan Normal University, Kunming, China; 2 School of Economics and Management, Kunming University, Kunming, China; Northeastern University (Shenyang China), CHINA

## Abstract

Ecological science focuses on the structure and function of the natural environment. However, the study of ecological environments primarily focuses on single-element research and lacks a comprehensive perspective. To examine ecological environmental trends on different scales, the present paper selected Yunnan Province as the study area. Chemical oxygen demand, rocky desertification, forest coverage, natural disaster data and spatial analysis methods were used to obtain the ecological environmental characteristics of each county and construct a comprehensive evaluation method of the ecological environment. The present paper revealed that the environmental capacity in Yunnan Province was at a moderate level, the ecological environment fragility was remarkable, the significance of the ecological environment was very high, natural disasters occurred frequently, and spatial differentiation between ecological environments was obvious. The province may be divided into three functional areas: the comprehensive-balanced area, the efficiency-dominated area and the environment-dominated area. Central Yunnan was a key development zone and the main area for the manufacturing and service industries, which were built as a modern industrial system in Yunnan Province. The ecological environment in northwestern Yunnan and southern Yunnan is of high significance, and this region was an ecological environment protection area that was important area for the construction of the modern agricultural system in Yunnan Province. To achieve sustainable development of the ecological environment, the spatial characteristics of the ecological environment must be determined at the county scale.

## 1 Introduction

The development of the world economy has exacerbated problems with the ecological environment since the 1990s. Scholars believe that the cost to the ecological environment of achieving economic growth is increasing, which has led to serious deterioration of the ecological environment that continues to intensify. The ecological environment has become a focus of political and academic concerns. Considerable ecological environment research was performed in the last 30 years, and this field has grown rapidly in the last 20 years. Research results are primarily published in journals, dissertations, conference papers and monographs. Research on the ecological environment is systemic and represents an important basis for the development of sustainable resource utilization, economic and social planning and ecological environment protection.

Research on the ecological environment primarily focuses on analyses of the water environment, atmospheric environment, soil environment, engineering technology, environmental protection technology, policy guidance, and industry technical guidance and determining solutions to typical regional ecological environment issues [1]. Many research methods were developed for ecological environments, including correlation analyses [2], environmental quality evaluations [3–7], comprehensive evaluation methods [8], fuzzy rule–based systems [9], neural network and gray models [10–12], analytic hierarchy processes [13], remote-sensing ecological indexes [14], GIS raster analysis [15], cellular automata [16], Google Earth Engine [17], geodetectors [18], and cellular automata [19]. High-tech methods, such as remote sensing, geographic information systems and global positioning systems, were used in ecological environmental evaluations and played a significant role. Moreover, “3S” technology has great potential and bright prospects. More attention focused on the remote-sensing inversion of parameters based on an in-depth quantitative analyses of environmental factors in developed countries. However, developing countries focused more on information acquisition and comprehensive quality evaluation applications at the macro level. The present research may be defined according to the following categories: acquisition of remote-sensing information on the ecological environment, establishment of an index system for assessment, evaluation of objects at different scales, exploration of methodologies and modeling methods for the assessment and determination of the assessment purpose and associated philosophy [1].

It is evident that recent developments in the geotechnologies of GIS and remote sensing have had a substantial impact on ecological environment research in the second decade of the 21st century and provided spatial data and associated information to enable further understanding of ecological systems [20]. Remote sensing has been widely used as a source of eco-environmental information for ecological research [21, 22]. For example, studies on biodiversity often sought to derive information on variables such as species richness and tried to facilitate biodiversity monitoring activities [23]. Many studies used land cover data, often as a surrogate for data on habitat type, and frequently exploited the temporal dimension of remote sensing to monitor land cover dynamics [24–26]. Changes in land use/land cover are key determinant factors among a broad range of land surface parameters (e.g., land surface temperature, evapotranspiration, and runoff) [24, 26]. Recent important developments in GIS include rapid growth in the number and variety of geographical data sets, finding new ways to store, process, and transmit these data sets, new forms of visualization and statistical/mathematical modeling. GIS has been a component of a diverse array of studies, and one popular theme is the linking of landscape patterns to a range of ecological variables. Species distribution modeling has become even more popular in recent years, especially given its role in predicting the impacts of variables and characterizing ecological niches [27–29].

Remote sensing and GIS have ensured their continuing input and significance in the field of ecological environments [20]. Satellite remote-sensing earth observation systems are widely used to evaluate ecological environments and have become an important part of ecological environment research [13]. Various remote-sensing indexes are used to monitor and evaluate forest [30], grassland [31], city [32, 33], river [34] and watershed ecosystems [35, 36]. A remote sensing-based ecological index was developed specifically for monitoring and assessing ecological environment changes. The index combines four evaluation indicators, namely, vegetation, humidity, land surface temperature and soil, and represents the four major ecological elements of greenness, humidity, heat, and dryness, which are important ecological environment indicators that are frequently used in assessing regional ecology [37–39]. The remote -sensing ecological index reflects the changes in ecological environmental quality more effectively than the ecological index [40]. The Google Earth Engine (GEE) cloud platform is the most advanced platform for geographic information data analysis and visualization. It stores a large number of historical images and geographic databases. This platform allows users to perform testing and development and preview the results in real time. It overcomes the low efficiency problems associated with local downloads, storage, and preprocessing and uses Google’s powerful computing capabilities to analyze and process a variety of environmental and social data [16]. With these advantages, GEE has been widely used to map land cover types and associated changes over large areas [41], perform data fusion [42, 43], act as a geodetector [44, 45], and investigate ecological environments [46–48].

Research indicated significant spatiotemporal heterogeneity between economic development and the ecological environment [49]. Ecological environment assessment is an important basis for eco-environmental protection and sustainable development [50]. Eco-environmental geography is centered on the interactions and relationships of the biogeophysical environment with human societies. Scholars are interested in environmental sustainability [51], ecological environment quality [52], ecological environment carrying capacity [53, 54], ecological footprint [55, 56], environmental capacity, fragility, significance, and natural disaster risk. These assessments are used to examine the major areas: environmental resource management, biodiversity conservation amid development and social change; climate change adaptation, natural hazards, vulnerability, and resilience; urban environmental systems; multiscale deforestation and reforestation; food, agriculture, environment, and health; and energy and society transitions [35–57].

Water environmental capacity is defined as the maximum amount of pollutants that a water body can hold and still meet water quality standards [58, 59]. Water environmental capacity is an important index for the management of water resources and environmental quality. Environmental impacts are often quantified based on whether water quality meets certain requirements in critical river locations, such as sites near drinking water sources that are important to human health. Various approaches were proposed to estimate the water environmental capacity ranging from simplified to complicated process-based models by mathematically representing the physical and chemical processes [60]. For example, GIS was used to provide a spatial analysis of the water environmental capacity calculation results [61, 62].

Research on the fragility of the ecological environment began in the 1960s. Research on ecological fragility has been a hot topic ever since 2000, and there were more empirical studies than theoretical studies. Studies in the early period used the city as the data unit. After 2000, a boom of theoretical reviews and initial signs of comprehensive research appeared, and the Grid GIS came into being. In response to requests from governments to show how the environment provides challenges and opportunities for human development, Global Environmental Outlook: Environment for Development gave the concept of vulnerability a central place. Vulnerability is the outcome of multiple stressors and multiple actors in multiple contexts that may occur at various spatial and time scales [63]. Eco-environmental vulnerability is primarily exaggerated by anthropogenic activities via land use/land cover changes and further enhanced by natural processes including disasters [64]. Remote sensing is a unique tool to provide complete and continuous land surface information at different scales, which may be used for eco-environment analysis. This technique is a baseline for highly accurate ecological condition mapping and monitoring and may be used for decision making, management and sustainable development [65, 66]. GIS technology provides data, modeling and technical support for the fragility of the ecological environment and performs data analyses and visualization. All datasets and mapping procedures may be processed in GIS using simple but powerful analyses procedures despite dealing with various complex environmental issues [67]. Geographically weighted principal component analysis can effectively quantifies environmental vulnerability [68, 69]. The use of global datasets related to eco-environmental vulnerability evaluation was primarily applied or limited to regional, provincial, and national scales. The largest fraction of the very high vulnerability level of continents is attributed to Asia (74.6%) followed by Africa (19.6%). National-scale analysis shows that China and India are the most vulnerable in Asia and the world [70]. Humans can improve the original highly vulnerable ecological environment via positive activities. Increasing ecological vulnerability areas should prohibit all acts that damage the ecological environment and improve the vulnerable ecological environment through positive human activities [71].

The ecological importance showed spatial heterogeneities across and within the regions. Protected area zoning has become an effective tool in recent decades to divide regions into different zones with different protection and development strategies, and it has considerably contributed to biodiversity conservation and the harmonious coexistence of multiple activities [72]. For the importance evaluation of regional ecological space, scholars primarily focused on ecological sensitivity and ecological suitability. Current studies on ecological evaluation primarily focused on the aspects of natural ecology, and the results did not reflect the spatial characteristics of regional ecosystems and maintaining ecological security. The ecological importance evaluation of regional space emphasizes the harmonious development between production space, living space, and ecological space, focuses on the symbiosis between humans and other organisms, and maintains the natural foundation of urban development using applied principles in ecology. Based on the analysis of ecological characteristics, ecological importance evaluation examines the spatial distribution of regional ecological importance and provides measures for preventing ecological security issues from regional development and construction [73–76]. A GIS-based approach proposes the results of the explicit and feasible multiscenarios, which facilitates the effective management of ecological space. The results show the spatial characteristics of eco-space for maintaining water security, biodiversity, disaster protection and recreation [74–76].

The study of ecological risk assessment started in the 1970s. Ecological risk assessment is a powerful tool for quantifying ecological risk. Ecological risk assessment calculates the possible ecological damage arising from exposure to one or more pollution components [77]. Present research on ecological risk assessment primarily focused on the following two aspects: large-scale and multilevel ecological risk assessments around regions, watersheds, coastal areas, and land use; and the establishment of a risk evaluation index system and evaluation standards, ecological risk assessments such as major ecological event assessment, landscape risk assessment, and species safety assessment [78]. Assessment of ecological risk primarily covers hazard assessment, exposure assessment, receptor analysis and risk characterization. Studies concentrated on the spatial distribution, temporal change, quantitative characteristics, spatial differentiation and regional division of ecological risks [79–81].

Research results on the ecological environment are not uncommon. However, this research is basically performed in a specific area and specific year. Therefore, the results reflect only the basic situation of the local area in that year and primarily focus on the study of a single element of the ecological environment. These studies are static. Therefore, the temporal and spatial changes in the ecological environment are difficult to identify. The present paper selected Yunnan Province as the research area to perform a spatial analysis of the ecological environment. The geographical environment of Yunnan Province is highly fragmented, and a single-element evaluation cannot provide a comprehensive explanation of the spatial differentiation characteristics of the environment. In light of the current status of research focusing on a single element of the ecological environment, such as environmental capacity, which can identify the water quality and water pollution status of an area, fragility reflects the sensitivity of the regional ecological environment to the outside world. Significance identifies locations for ecological environment protection, and hazards provide early warnings for earthquake prevention and disaster reduction. The present paper used Yunnan Province as the spatial scale. Focusing on ecological environment theory, this paper constructs a comprehensive evaluation system of the ecological environment and explores the spatial differentiation characteristics of the ecological environment in Yunnan Province to provide a spatial and practical basis for ecological environment research.

This paper combines environmental capacity, fragility, significance, hazards and GIS and innovatively proposes a comprehensive research method. Section 2 shows the calculation process for the comprehensive research method. Section 3 presents the application of this method in the study of the ecological environment in Yunnan Province. Section 4 provides suggestions for the sustainable development of the ecological environment in Yunnan Province. Section 5 presents the conclusion.

## 2 Study material

### 2.1 Study area

**Fig 1:**
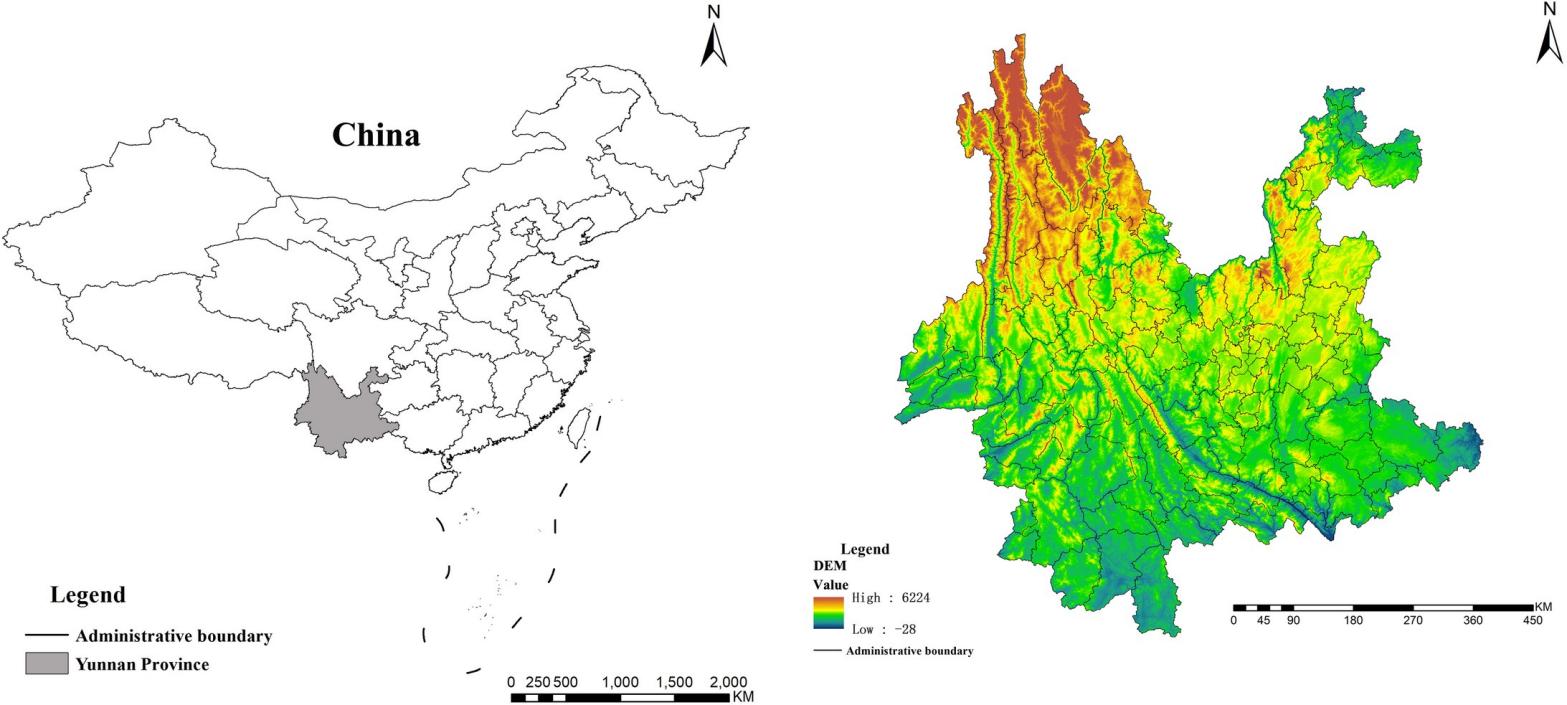
Location of Yunnan Province. (Data source: http://bzdt.ch.mnr.gov.cn/index.html).

As shown in Fig 1, the Yunnan Province is located in the border regions of China (21°8′-29°15′N, 97°31′-106°11′E). Yunnan Province has 16 cities, 8 ethnic autonomous prefectures, and 124 counties. With a gross area of 394,100 km^2^, the area of Yunnan Province accounts for approximately 4.11% of the area of China, and mountain areas account for more than 84% of the total area of Yunnan. The total population was 48.3 million in 2018, which accounted for approximately 3.46% of the country. The population density was 121.8 people/km^2^, and the GDP was 1,788,112 million yuan, which accounted for approximately 2% of the GDP of China. Yunnan Province is located in the Chinese frontier area and has the second largest forest area in China. It is an important area for maintaining the stability of the overall ecological environment of China. The forests in this area are mostly distributed in the source area of the river system and the upper and middle reaches of the river basin. The quantity, quality and distribution of the forest are closely related to the ecological environment of a partial area or the entire river basin. The concentrated distribution areas of forests represent the last habitats of the main wild animal and plant resources in Yunnan. As a typical representative border region in China, the supporting capacity of the ecological environment has changed significantly with the development of the economy in mountainous areas, and regulating the deterioration of the ecological environment foundation has become increasingly difficult. Future prospects are not optimistic. Ecological environmental protection has significant benefits in the contemporary era and for future generations. As a source of large rivers in southwestern China, Yunnan Province should place greater emphasis on the ecological environment. Therefore, the maintenance function and social value of the ecological environment in Yunnan Province are far greater than the economic value of these environments. Environmental protection and development in this area must be considered from the perspective of ecological security of the entire river basin and country and the sustainable development of the social economy.

### 2.2 Data and methods

#### 2.2.1 Data

Chemical oxygen demand (COD) and ammonia nitrogen capacity data for rivers were obtained from the *Major Function-Oriented Zone Planning in Yunnan Province* (http://www.yn.gov.cn/zwgk/). The soil erosion and rocky desertification data were obtained from the *Report on the State of Yunnan Provincial Environment* (http://sthjt.yn.gov.cn/hjzl/). The ecological protection data were obtained from the *List of Natural Reserves in Yunnan Province* (http://sthjt.yn.gov.cn/zrst/). The earthquake and debris flow data were obtained from the *Yearbook of Disaster Reduction in Yunnan Province* (http://yndzj.gov.cn/yndzj/sy1/index.html), Yunnan Earthquake Prevention and Disaster Reduction Bureau, Department of Ecology and Environment of Yunnan Province. Relevant data were standardized.

#### 2.2.2 Methods

Humans are the main body in the ecological environment, and other living creatures and nonliving objects are a complex of environmental elements (e.g., terrain, climate, soil, and vegetation). Traditional single-element assessment methods for the ecological environment focus more on analyzing the spatial and temporal distribution characteristics of a single element. Under diversified social development, ecological environment research has obvious comprehensive and holistic characteristics, and the interaction between basic elements of the ecological environment and their associated influence is becoming increasingly obvious. Combined with the objective requirements of developing scientific concepts, ecological environment research aims to overcome the limitations of traditional single-element assessment methods. This paper innovatively constructs a multielement comprehensive evaluation method and emphasizes the possibility of coordinating the balance of the internal system with the external environment.

According to the special geographical environment of Yunnan Province, the following comprehensive evaluation system model of the ecological environment is proposed:

Comprehensive evaluation index system of the ecological environment (*L*) = environmental capacity index (*E*)+fragility index (*Y*)+significance index (*P*)+hazard index (*DH*).

*(1) Water environmental capacity index system*. Japanese scholars proposed the concept of environmental capacity, and the term is rarely used by European and American scholars. Chinese research on environmental capacity began in the 1970s. Environmental capacity is the maximum load of pollutants that the environmental system can accommodate within a certain time frame, and it is the upper limit of the carrying capacity according to the resource and environmental conditions at the development scale. This paper calculated the water environmental capacity using COD and ammonia nitrogen data for the river section at the county level in Yunnan.

$$E_{cod}=C_{cod}\cdot Q\cdot \alpha {–q}_{\mathrm{cod}}$$ (1)

$$E_{n}=C_{n}\cdot Q\cdot \alpha -q_{n}$$ (2)

where *E*_*cod*_ is the environmental capacity of COD (t/a), *C*_cod_ is the concentration of COD (mg/L), *q*_*cod*_ is the amount of COD flowing into the river (t/a), *E*_*n*_ is the environmental capacity of ammonia nitrogen (t/a), *C*_n_ is the NH_3_-N concentration (mg/L), *q*_n_ is the amount of NH_3_-N flow into the river (t/a), Q is the average water volume (t/a), and α is the water resource utilization coefficient, which has a value of 0.4.

*(2) Fragility index system*. Scholars determined that sensitivity and instability were the most important characteristics of ecologically fragile areas. The fragility of the ecological environment means that the ecological environment is affected by external interference and factors beyond its own adjustment range. Therefore, the ecological environment shows varying degrees of sensitivity to external interference. The ecological environmental fragility coefficient of Yunnan Province was calculated using the following formula:

$$Y=1-\sum P_{i}\cdot W_{i}$$ (3)

where *Y* is the ecological environment fragility coefficient, *P*_*i*_ is the dimensionless value of the *i*-th index, and *W*_*i*_ is the weight value of the *i*-th index.

*(3) Significance index system*. Ecological significance is an evaluation index for dividing restricted and prohibited development zones within the main functional zone of the province. Many scholars evaluated the importance of ecosystems by analyzing their composition and quantitative service value within a certain national space. Based on the area of nature reserves above the provincial level, this paper calculated the proportion of ecologically important areas in each county.

$$P_{i}=\frac{N_{i}}{T_{i}}\times 100\%$$ (4)

where *P*_*i*_ is the ratio of the assessment of regional biodiversity protection areas and ecologically important areas, *N*_*i*_ is the area of an important ecological environment, and *T*_*i*_ is the total area of the region.

*(4) Hazard index system*. The single-element evaluation of natural disaster risk is thorough, and the method of risk assessment shifted from qualitative analysis to comprehensive quantitative evaluations that combine natural and social science. This paper used Graham’s method to evaluate natural disasters.

$$D_{i}=L_{i}\times E_{i}\times C_{i}$$ (5)

where *D*_*i*_ is the single-element risk index of natural disasters, *L*_*i*_ is the average probability of occurrence of disasters, *E*_*i*_ is the time of recipient affected by the disasters, and *C*_*i*_ is the possible loss caused by disasters.

$$DH=\sum_{i=1}^{n}\lambda \times D_{i}$$ (6)

where is the natural disaster risk index, *λ* is the disaster index weight, and *D*_*i*_ is the disaster risk index.

*(5) Comprehensive ecological environment evaluation index system*. Using 124 counties as the basic geographical unit, a comprehensive value measurement algorithm was adopted after standardizing the water environmental capacity index (E), fragility index (Y), importance index (P) and hazard index (DH). The score range of each indicator was no more than 10.

$$y_{ij}=\frac{x_{ij}-\min x_{ij}}{\max x_{ij}-\min x_{ij}}\;(1\leq i\leq m,1\leq j\leq n)$$ (7)

$$y_{ij}=\frac{\max x_{ij}-x_{ij}}{\max x_{ij}-\min x_{ij}}(1\leq i\leq m,1\leq j\leq n)$$ (8)

$$L=100\cdot \sum (K_{i}\cdot A_{i})\;(i=1,2,3\ldots ,n)$$ (9)

where L is the comprehensive index for evaluating the ecological environment of the region, *K*_*i*_ is the index weight, and *A*_*i*_ is the index score.

## 3 Evaluation results

### 3.1 Moderate environmental capacity

Based on the minimum value of the remaining amounts of COD and NH3-N in the river section of each county in Yunnan, the results of the study showed that the ecological environment capacity was moderate in Yunnan Province, with 91 counties above the moderate level and 33 counties below the low level. Using the natural classification method, the results using Arcview (ArcGIS10.2 https://desktop.arcgis.com/zh-cn/) data show that the water environmental capacity in Yunnan may be divided into 5 levels, which are shown in Table 1.

**Table 1:** Classification of the water environmental capacity in Yunnan Province.

Water environmental capacity (t/a)	Level	City			
<2000	smaller	Kunming			
2000-3500	small	Yuxi	Chuxiong	Diqing	
3501-5000	medium	Lijiang	Xishuangbanna	Dali	Qujing
5001-6500	big	Dehong	Zhaotong	Honghe	Baoshan
>6500	bigger	Wenshan	Nujiang	Lincang	Pu’er

**Fig 2:**
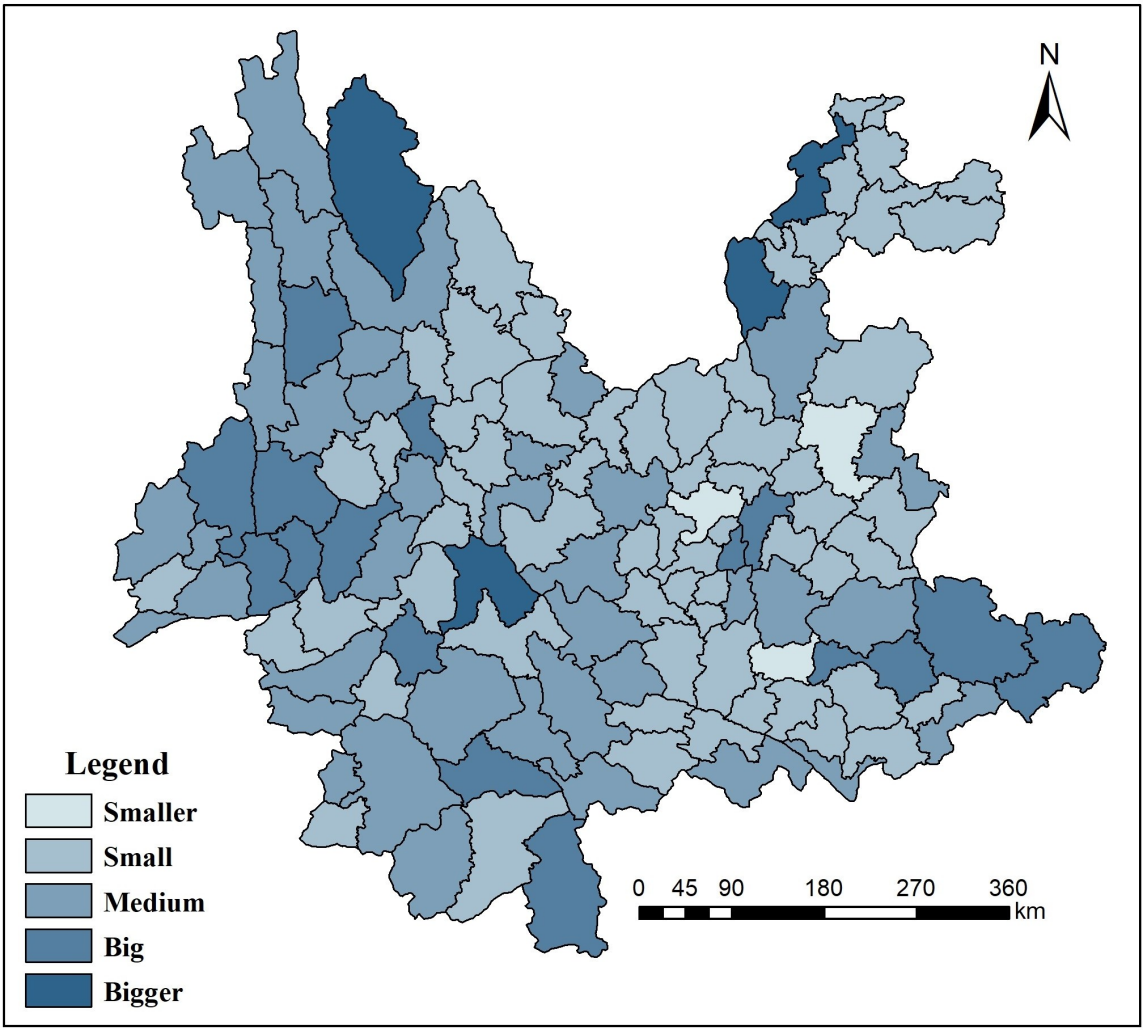
Spatial differentiation of the water environmental capacity in Yunnan Province.

According to Table 1, the lowest water environmental capacity was observed over an area of 21,473 km^2^, which accounted for approximately 5.5% of the total area of Yunnan Province. Due to urban expansion and industrial development, Kunming had serious water pollution and excessive water resource consumption, especially in Dianchi Lake. Fig 2 shows that the area with a small water environmental capacity was 56,069 km^2^, which accounted for approximately 14.2% of the total area. Yuxi and Chuxiong have experienced problems in recent years caused by the use of surface water by the new industrialization and urbanization of central Yunnan industrial clusters. The area of drainage basin in Diqing is small. The area with medium water environmental capacity was 104,000 km^2^, which accounted for approximately 26.4% of the total area. Dali and Lijiang had less industrial pollution, more plateau lakes, better water quality, and greater water volume. Surface rivers are more distributed in Qujing. Xishuangbanna has more precipitation. The area with a large water environmental capacity was 87,114 km^2^, which accounted for approximately 22.1% of the total area. There are many rivers in this area, and the emission amount is small. The area with a higher water environmental capacity was 116,800 km^2^, which accounted for approximately 29.6% of the total area. The industrial development speed in this area is slow, and the rivers in the area are dense. Therefore, the water storage capacity of the surface is relatively strong.

### 3.2 Relatively fragile ecological environment

The ecological environment fragility evaluation index includes the cause and performance characteristics of the fragile ecological environment. According to the cause-result formation process of the fragile ecological environment and specific characteristics of the county ecological environment in Yunnan Province, the principal component analysis method was used to construct the fragility evaluation index system, as shown in Table 2.

**Table 2:** Ecological environmental fragility index system.

Indicator code	Index	Weight
Natural cause indicator		0.50
*X*_*1*_	precipitation	0.10
*X*_*2*_	forest coverage	0.15
*X*_*3*_	arable land area per capita	0.10
*X*_*4*_	proportion of protected area	0.15
Results performance index		0.50
*X*_*5*_	GDP per capita	0.10
*X*_*6*_	population density	0.15
*X*_*7*_	disposable income of urban residents	0.05
*X*_*8*_	per capita net income of farmers	0.05
*X*_*9*_	Engel coefficient	0.15

Greater fragility indicates a more fragile ecological environment and worse stability of the system. Lower fragility indicates a less fragile ecological environment. As shown in Table 3, the results of the study indicated that the ecological environment in Yunnan Province was relatively fragile and may be divided into 4 levels: 38 counties were mildly fragile, 24 counties were moderately fragile, 40 counties were strongly fragile, and 22 counties were extremely fragile.

**Table 3:** Ecological environmental fragility levels in Yunnan Province.

Fragility coefficient	Level	City				
<0.4	Mildly fragile	Kunming	Chuxiong	Yuxi	Qujing	
0.4-0.5	Moderately fragile	Dali	Baoshan	Lijiang	Xishuangbanna	
0.5-0.65	Strongly fragile	Lincang	Dehong	Pu’er	Honghe	Nujiang
>0.65	Extremely fragile	Zhaotong	Wenshan	Diqing		

**Fig 3:**
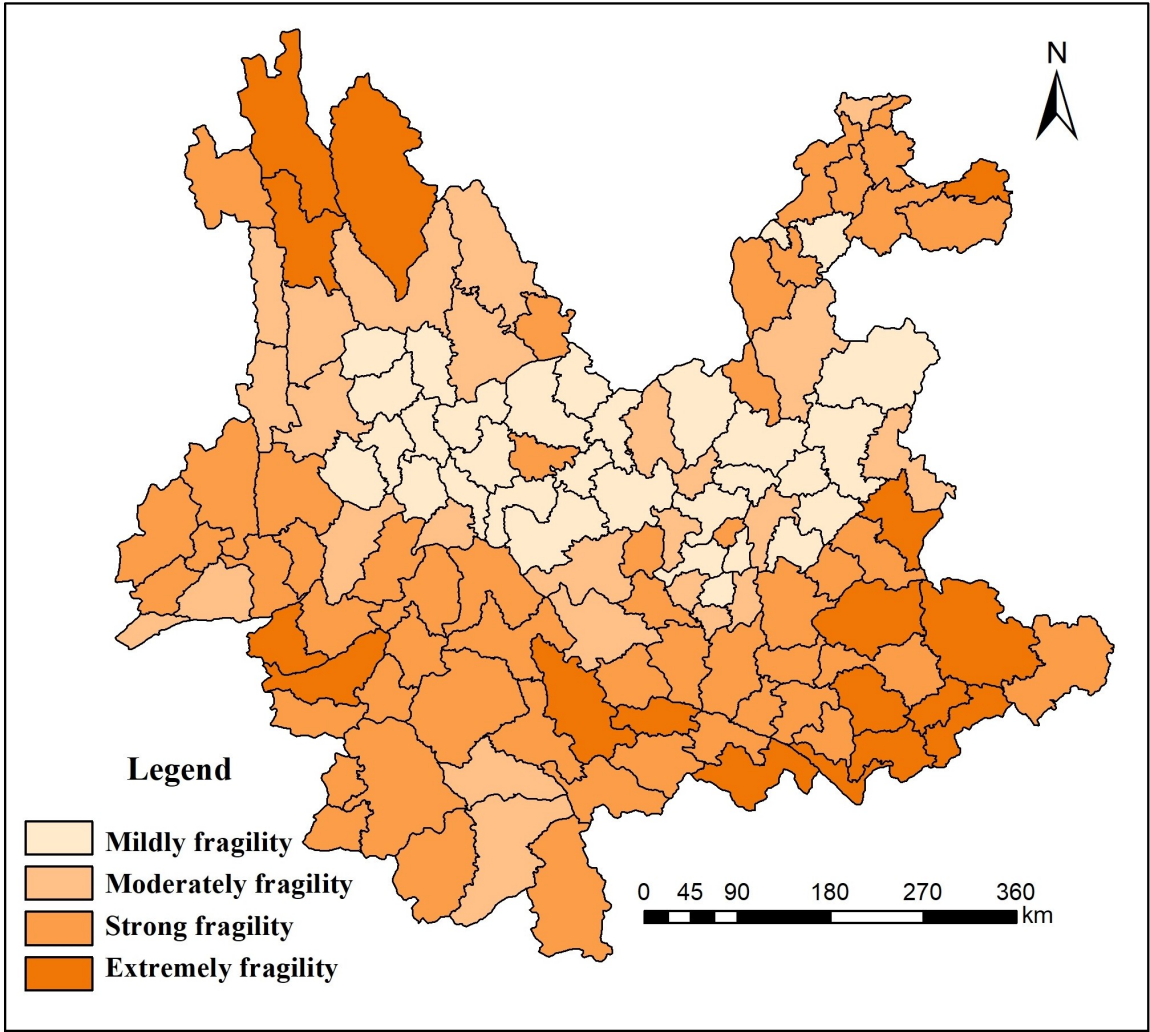
Ecological environment fragility assessment map of Yunnan Province.

**Fig 4:**
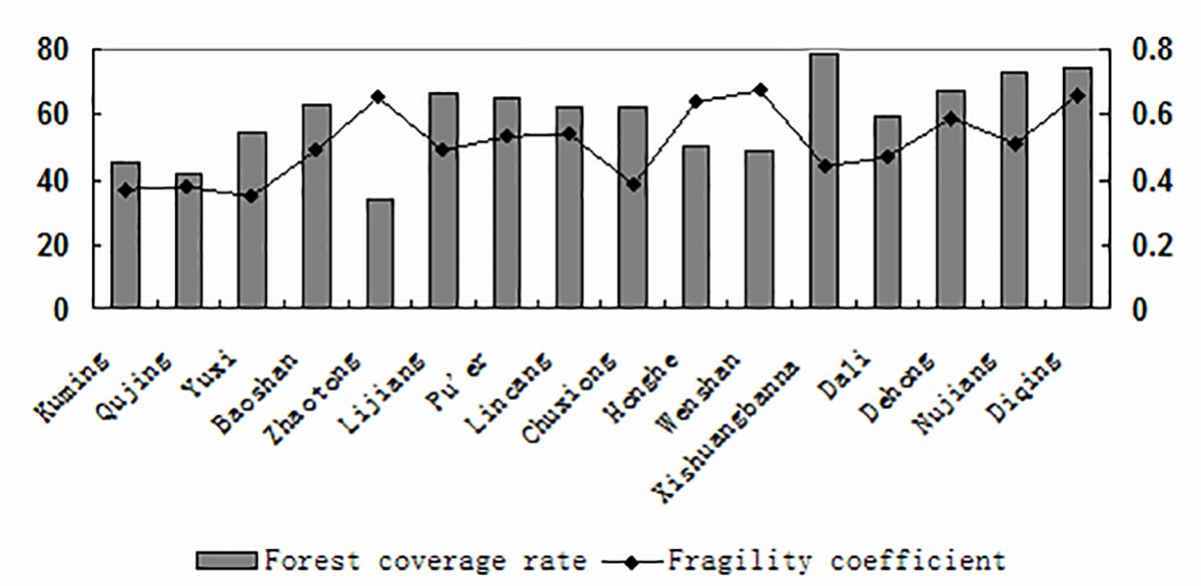
Forest coverage rate and fragility coefficient of Yunnan Province.

As seen in Fig 3, the mildly fragile regions were distributed in the urban agglomeration of central Yunnan, with an area of 95,600 km^2^, which accounted for approximately 24.5% of the total area of Yunnan Province. Fig 4 shows that Kunming, Qujing, Yuxi and Chuxiong were the most economically developed areas in Yunnan Province. The regional resources and environment of these areas were superior, the population concentration conditions were good, and the ecological environment resistance ability was strong. The moderately fragile region had an area of 90,015 km^2^, which accounted for approximately 23.1% of the total area. The forest coverage rate was high, the vegetation was relatively stable, the degree of human disturbance was low, and the ecological recovery ability was good. The strongly fragile region had an area of 129,000 km^2^, which accounted for approximately 33.1% of the total area. The geological structure of this type of area was complex and changeable, geological disasters occured frequently, and vegetation restoration was relatively difficult. The extremely fragile region had an area of 79,130 km^2^, which accounted for approximately 20.3% of the total area. The vegetation in this type of area was seriously damaged by humans, the damage caused by natural disasters was severe, the ecological environment itself had poor stability, and ecological restoration was rather difficult.

### 3.3 Relatively significant ecological environment

Ecological significance is the basis for the provincial division of forbidden development zones. There are 157 nature reserves of different levels in Yunnan Province, and they have a total area of 28,253 km^2^, which accounted for 7.4% of the total land area of the province. Twenty reserves are at the national level, and 38 reserves are at the provincial level. The area of nature reserves above the provincial level was 21,260.21 km^2^, and the ecological environment was comparatively important.

**Fig 5:**
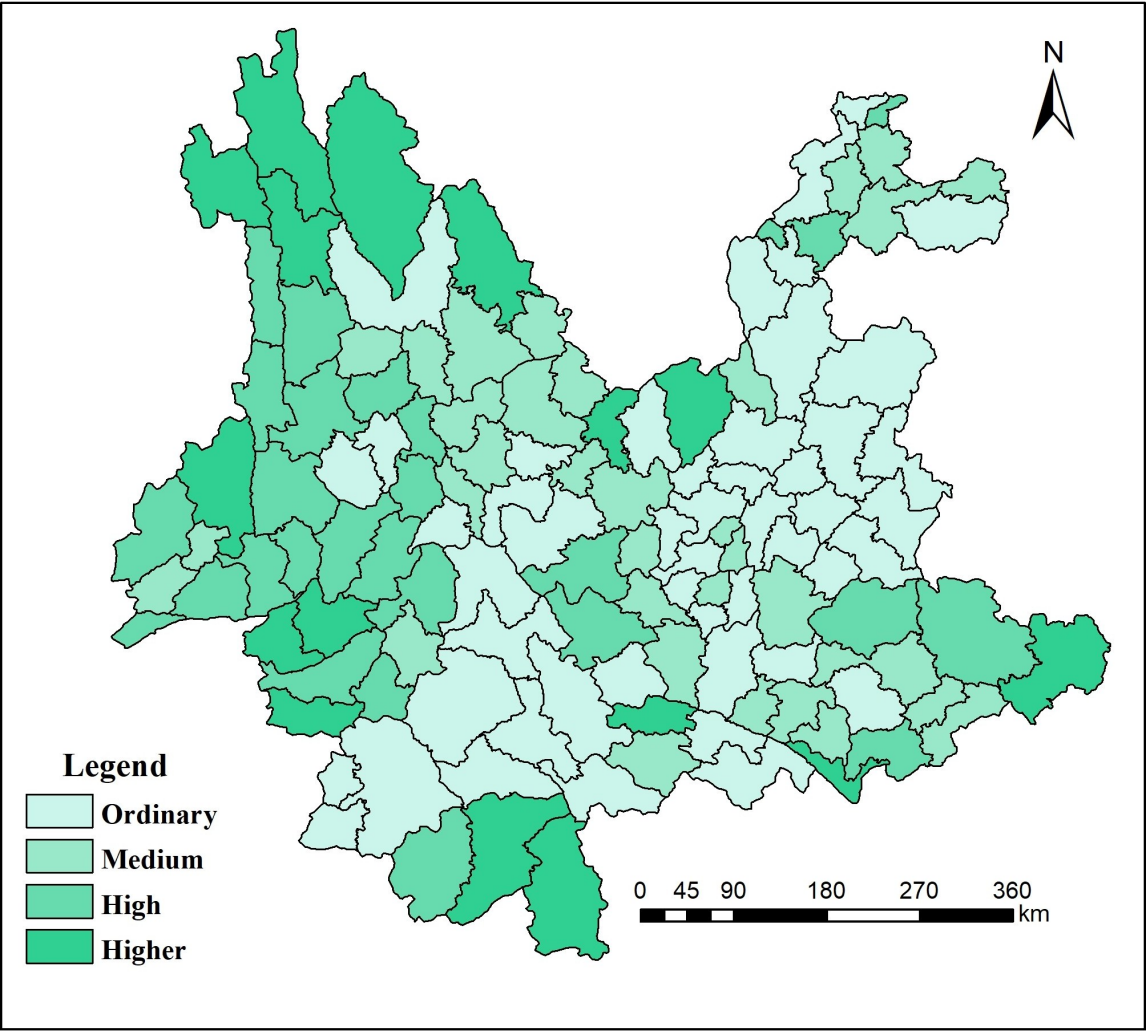
Evaluation map of the significance of the ecological environment in Yunnan Province.

The present paper used the spatial analyst module of GIS to generate an evaluation map of the significance of the ecological environment in Yunnan Province, as shown in Table 4. The results showed that the ecological significance of 124 counties in Yunnan Province may be divided into 4 levels, with 90 counties of moderate significance and 34 counties of low significance. The area of low significance was approximately 103,000 km^2^, which accounted for approximately 26.5% of the total area, and the area of moderate significance was approximately 114,000 km^2^, which accounted for approximately 29.2% of the total area. The area of high significance was approximately 93,000 km^2^, which accounted for 23.84% of the total area, and the area of higher significance was approximately 75,400 km^2^, which accounted for approximately 19.3% of the total area, as shown in Fig 5.

**Table 4:** Proportion of nature reserves in Yunnan Province.

City	Kunming	Qujing	Chuxiong	Yuxi	Zhaotong	Dali	Lincang	Honghe
Nature reserve (km^2)^	237.31	1669.50	843.13	241.00	656.64	1165.81	2022.33	1396.29
Proportion(%)	1.11	4.94	2.88	1.58	2.85	3.96	8.26	4.24
City	Wenshan	Xishuangbanna	Baoshan	Lijiang	Diqing	Dehong	Puer	Nujiang
Nature reserve (km^2)^	904.12	2683.76	4131.22	406.60	3148.41	516.51	478.69	758.94
Proportion (%)	2.80	13.62	21.04	1.29	13.19	4.48	1.05	5.16

### 3.4 Natural disasters occur frequently

According to the basic data of natural disasters in Yunnan Province, a risk assessment system of natural disaster elements was constructed, as shown in Table 5.

**Table 5:** Risk assessment system of natural disaster elements.

Types of disaster	Index	Weight
Geological hazard	landslide	0.20
	debris flow	0.20
Earthquake hazard	earthquake magnitude	0.30
	anti-seismic capability	0.15
	emergency relief capability	0.15

**Fig 6:**
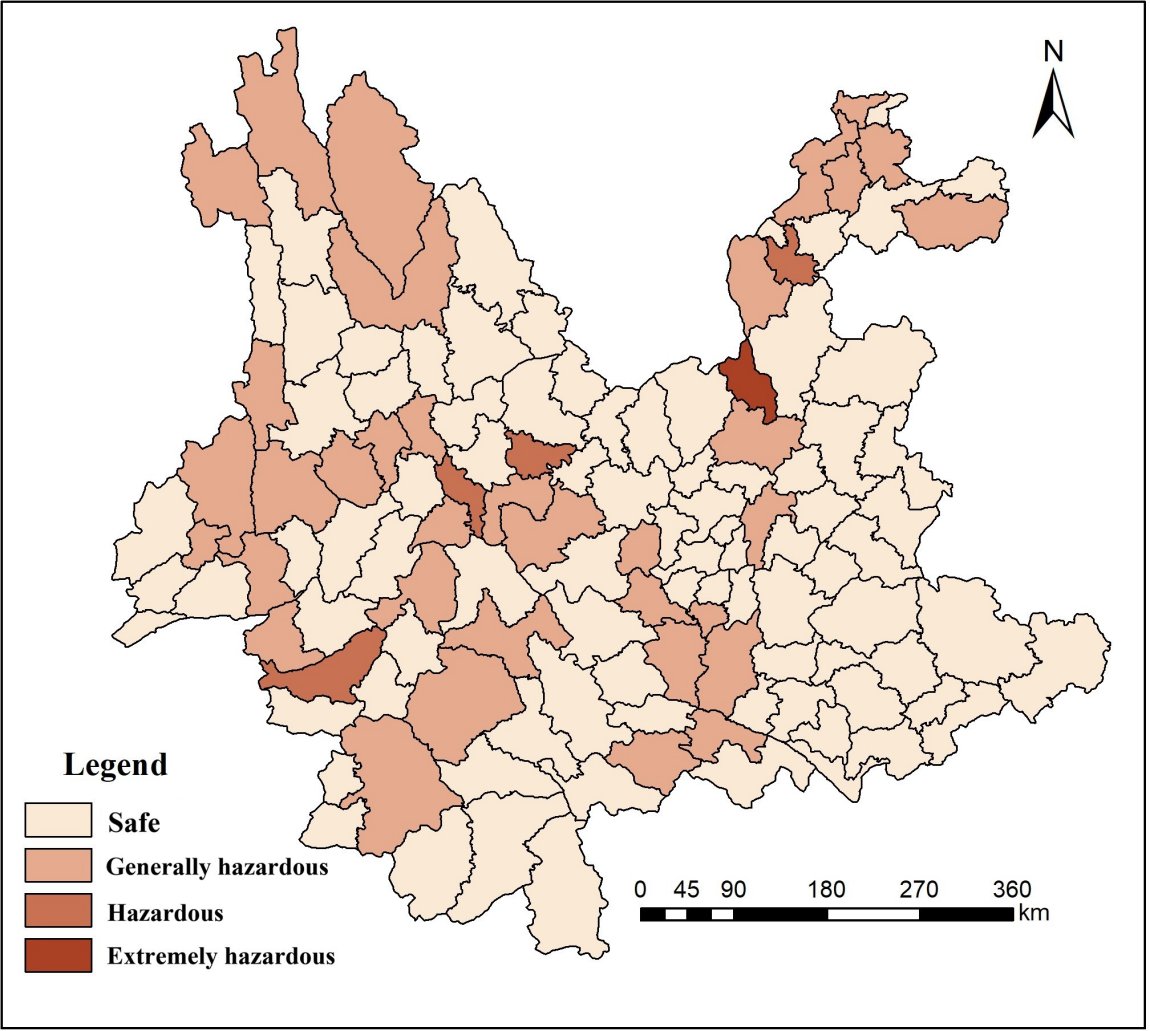
Risk assessment of geological disasters in Yunnan Province.

As seen in Fig 6, the natural disaster evaluation results calculated using Graham’s method showed that Yunnan Province is a region with frequent natural disasters and the 124 counties may be divided into three levels. (1) Extremely hazardous area: Dongchuan, with an area of 1,856.79 km^2^, which accounted for 0.48% of the total area. Dongchuan is located in the Xiaojiang fault zone. Therefore, seismic activity occurs relatively frequently, and surface geological fragmentation is high. It is a region with little rain, and long-term mining caused serious ecological degradation. Therefore, the restoration costs are extremely high. (2) Hazardous area: Gengma, Midu, Yao’an and Ludian, with an area of 8,730 km^2^, which accounted for 2.24% of the total area. Earthquake disasters and landslides and debris flows occurred in Gengma and Midu. The earthquake disasters in Yao’an and Ludian were very obvious in recent years. (3) Generally hazardous area: Yuxi, Chuxiong, Zhaotong(except Ludian), Honghe, Pu’er, Baoshan, Lincang, Dehong, Dali, Lijiang and partial areas of Diqing, covering an area of 142,397 km^2^, which accounted for approximately 36.51% of the total area. Earthquakes and debris flows occur in Zhaotong. Landslides and debris flows are seen frequently in Honghe, and earthquake disasters often occur in western Yunnan Province.

### 3.5 Obvious spatial differentiation of the ecological environment

As shown in Table 6, the present paper used the comprehensive value measurement algorithm to calculate the comprehensive scores of the ecological environmental quality of all counties in Yunnan Province. According to the calculation results, the paper divided the ecological environment of Yunnan Province into 3 types: comprehensive-balanced area, efficiency-dominated area and environment-dominated area.

**Table 6:** Comprehensive scores of the ecological environment quality of each city in Yunnan Province.

	City	*E* value	*Y* value	*P* value	*DH* value	Total score
Comprehensive balance area	Baoshan	0.1232	0.1688	0.1940	0.1474	63.33
	Xishuangbannan	0.0680	0.2156	0.1258	0.1895	59.89
	Nujiang	0.2910	0.1313	0.0060	0.0947	52.30
	Pu’er	0.1376	0.1500	0.0411	0.1684	49.72
	Lincang	0.1593	0.1219	0.0721	0.1158	46.91
Efficiency- dominated area	Qujing	0.0851	0.2766	0.0389	0.1940	59.45
	Chuxiong	0.0463	0.2719	0.0183	0.1263	46.29
	Kuming	0.0235	0.2910	0.0053	0.1053	42.51
	Yuxi	0.0581	0.1725	0.0087	0.1789	41.82
Environment- dominated area	Wenshan	0.0090	0.2859	0.0006	0.0842	37.97
	Dehong	0.0983	0.0750	0.0343	0.1474	35.50
	Lijiang	0.0519	0.0094	0.1215	0.1684	35.12
	Diqing	0.1282	0.0090	0.0176	0.1789	33.37
	Honghe	0.0677	0.1875	0.0291	0.0421	32.64
	Dali	0.1032	0.0281	0.0319	0.1263	28.96
	Zhaotong	0.0985	0.0188	0.0180	0.0060	14.13

Comprehensive-balanced area: Baoshan, Xishuangbanna, Qujing, Nujiang, Pu’er and Lincang. The natural geographical environment in these areas was good, and the ecological benefits and the environmental quality were relatively balanced. With a water environmental capacity of more than 6500 (t/a), Nujiang, Pu’er and Lincang are rich in water resources and have a high water environment capacity. Baoshan and Xishuangbanna have abundant biodiversity and higher stability of surface vegetation, and the ecologically important regions account for 21.04% and 13.62% of the whole area, respectively, which indicates that these cities have the highest ecological significance.

Efficiency-dominated area: distributed in the central Yunnan urban agglomerations, including Kunming, Qujing, Yuxi and Chuxiong. This type of area had the most developed economy, high resource utilization efficiency, relatively high technical level, good cultural environment, and ecological benefits that exceeded the environmental quality. Economic development created pressure on the ecological environment. Kunming (with a GDP of 520.69 billion yuan), Qu Jing (with a GDP of 201.34 billion yuan), Yuxi (with a GDP of 149.30 billion yuan) and Chuxiong (with a GDP of 102.43 billion yuan) are economically developed regions in Yunnan Province, and the economies of these cities ranked first, second, fourth and sixth in Yunnan in 2018, respectively. Kunming, Qujing, Yuxi and Chuxiong are neighboring areas, and these cities form the central Yunnan urban agglomeration. The GDP of the central Yunnan urban agglomeration accounted for 54.46% of the GDP of Yunnan Province, which made it the economic growth pole of Southwest China. The ecologies of Kunming, Qujing, Yuxi and Chuxiong showed limited fragility and strong resistance to external interference. The rapid development of urbanization and industrialization led to a backward environmental quality. The water environmental capacities of Chuxiong (3,071.41 t/a) and Yuxi (2,098.38 t/a) were relatively low. The water environment capacity of the Dianchi Lake Basin in Kunming is extremely low. Therefore, maintaining ecological benefits and improving environmental quality are key points for constructing ecological civilizations in Kunming, Qujing, Yuxi and Chuxiong.

**Fig 7:**
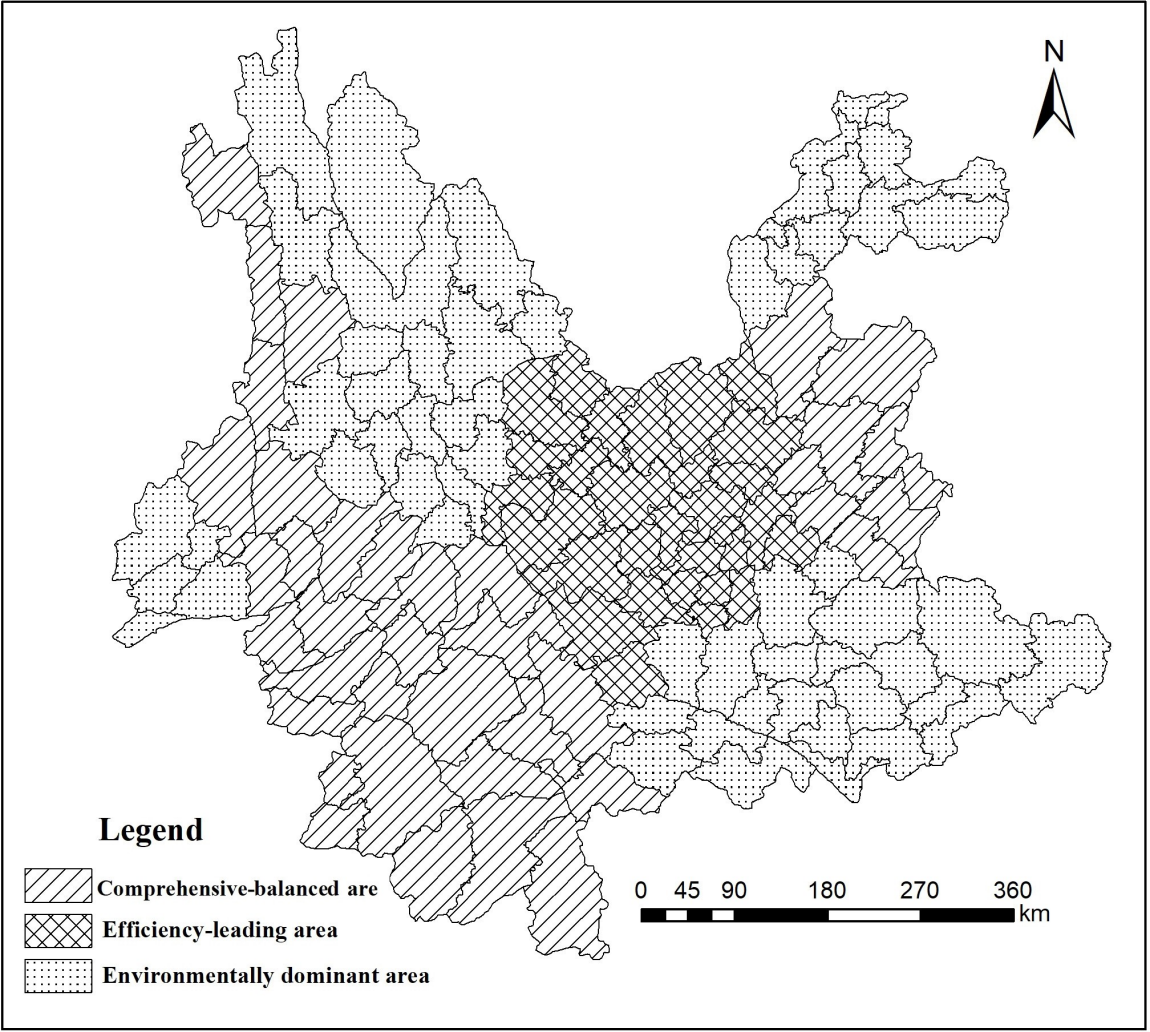
Ecological environmental zoning in Yunnan Province.

Environmentally dominated areas primarily included Wenshan, Dehong, Lijiang, Diqing, Honghe, Dali and Zhaotong. With good natural environmental quality, the sustainable development of these cities was dominated by environmental quality, moderate natural resource utilization rates, and low ecological benefits. The energy consumption per 10,000 yuan of GDP in these areas was not high, the proportion of the secondary industry was low, the proportion of the primary and tertiary industries was large, the GDP per capita was low, and the utilization rate of ecological resources was insufficient. These areas have many biodiversity-protected areas, and the ecological environment was important. The water environmental capacity was greater than 3500 t/a, the ecology was moderately fragile, and vegetation recovery was difficult, as shown in Fig 7.

From the perspective of the overall ecological environment, the ecological environment foundation of Yunnan Province was relatively good. However, due to the complexity of the mountainous geographical environment, challenges remain in improving the ecological environment of certain areas. An evaluation of the four indicators of environmental capacity, ecological fragility, ecological importance and natural disaster hazard indicated obvious spatial differences, with the ecological environment in the northwest and southwest areas of Yunnan Province being relatively good and the northeast and central areas of Yunnan relatively weak.

## 4 Conclusions

Ecological science is the study of the structure and function of the natural environment. The present paper used the relevant principles of ecology to address the current situation of single-element research on the ecological environment, constructed a comprehensive evaluation method of the ecological environment from the perspective of system theory, focused on the prefectural scale, and examined the ecological environment and spatial differentiation characteristics of Yunnan Province. This research has certain theoretical significance and provides a theoretical perspective and practical basis for the construction of ecological civilization space in Yunnan Province. Based on the overall ecological environment, the ecological environment foundation of Yunnan Province was relatively good. However, the geographical environments of mountainous areas are very complex. The environmental capacity, ecological environmental fragility, ecological environmental importance and natural disaster hazards indicators were evaluated, and we found obvious spatial differences, with the ecological environment in the northwestern and southwestern regions of Yunnan being relatively good and that the northeastern and central regions of Yunnan Province being relatively weak.

This study found that Yunnan had a moderate environmental capacity, a relatively fragile ecological environment, high ecological environment importance, and frequent natural disasters. The ecological environment had obvious spatial differences, which were divided into three functional areas: comprehensive-balanced area, efficiency-dominated area, and environment-dominated area. Ecological environmental research provides an important basis for the formulation of sustainable economic and social development plans and ecological environmental protection countermeasures. Although the ecological environment was mildly fragile in central Yunnan, this area is a key development zone and the main area for the manufacturing and service industries, which were built as a modern industrial system in Yunnan Province. The ecological environment in northwestern and southern Yunnan is of high significance, and this region is an ecological environment protection area and an important area for the construction of the modern agricultural system in Yunnan Province. According to the environmental capacity, the water quality of Shangri-La, Jingdong, Yongshan, Qiaojia, Tengchong, Baoshan, Dali, Lincang and Pu’er was good and the water pollution in Kunming Dianchi Lake was serious. According to the environmental fragility analysis, the ecological environments of Deqin, Shangri-La, Weixi, Weixin, Zhenkang, Gengma, Mojiang, Honghe, Hekou, Maguan, Wenshan, Malipo, Xichou, Qiubei, Guangnan and Luoping were most obviously affected by external influences. According to the significance analysis, the key areas of ecological environment protection in Yunnan Province included Deqin, Shangri-La, Weixi, Gongshan, Ninglang, Yuanmou, Tengchong, Zhenkang, Yongde, Cangyuan, Jinghong, Mengla, Honghe, Hekou, Luquan, and Funing. These locations had the most ecological environmental protection zones. The hazard analysis identified areas with frequent natural disasters and hidden hazards in Yunnan Province. For example, mudslides were more likely to occur in Dongchuan and earthquakes were more likely to occur in Ludian and Yao’an. Ludian experienced an earthquake in 2014, and Yao’an experienced an earthquake in 2009. Hazard research results have important reference value as disaster warnings.

As a hot research field, the ecological environment has obvious comprehensive and systematic characteristics. A comprehensive evaluation system for the ecological environment was constructed as an exploratory research method, and it showed regional characteristics. Particular emphasis should be placed on the selection of indicators for different research spaces in the future.

## 5 Policy implications

### 5.1 Ecological benefit improvements in environment-dominated areas

A number of methods are used in environment-dominated areas, including a new round of ecological civilization construction plans, the construction and improvement of a dynamic monitoring network for vegetation coverage, strict implementation of forestland reserves, promotion of forest planting and grassland planting, and scientific development of aboveground and belowground spaces to realize potential land uses and increase surface vegetation coverage. Wenshan, Dehong, Lijiang, Diqing, Honghe, Dali and Zhaotong could gradually improve the level of ecological benefits by enhancing the level of technology and management and increasing the utilization rate of ecological resources.

### 5.2 Environmental quality improvements for the comprehensive-balanced area

From the perspective of ecological environmental protection, the comprehensive-balanced area could set goals for sustainable economic and social development. Qujing should actively develop its light industry and service industry to prevent hidden dangers caused by the rapid development of highly energy-consuming industries. Baoshan, Xishuangbanna, Nujiang, Pu’er and Lincang could earnestly implement the target responsibility system and accountability system of environmental protection and actively perform paid use of pollution discharge rights and trading pilots. This work would emphasize that the development and protection of the comprehensive-balanced area should work together to improve environmental quality and capacity.

### 5.3 Strengthen the reform and transformation of efficiency-dominated areas

The resource-environment dependence path of economic development in efficiency-dominated areas is obvious, and the carrying capacity of the ecological environment hinders the speed of economic and social development. By promoting economic and social development transformation through "reform", Kunming, Yuxi, and Chuxiong are capable of breaking through the restriction of the ecological environment on economic and social development to change the traditional resource-dependent development model and transfer the mode of production to promote economic transition and realize the transformation of economic and social development and the ecological environment from "investment" to "reform".

### 5.4 Accelerating law-based governance of the ecological environment of the region

The comprehensive-balanced area, efficiency-dominated area and environment-dominated area should thoroughly implement sustainable development, fully promote law-based governance of the ecological environment, adhere to promoting environmental development, strengthen efforts to promote the ecological environment legal system, and create a good social atmosphere for protecting the ecological environment. The monitoring function of grassroots environmental protection organizations should be supported to provide guarantees for the sustainable utilization of the ecological environment.

## Supporting information

S1 Data (RAR)
